# Analysis of the *Salmonella enterica* serovar Typhimurium Chitobiose ( *chb* ) Operon

**DOI:** 10.17912/micropub.biology.002070

**Published:** 2026-05-08

**Authors:** Kristin Hobson, Melanie Higgins

**Affiliations:** 1 Biological Sciences, University of Alabama, Tuscaloosa, AL, US

## Abstract

*Salmonella enterica* serovar Typhimurium ( *S.* Typhimurium) is an intracellular pathogen that employs specialized virulence factors to survive and replicate within host cells, including a putative chitobiose ( *chb)* operon traditionally associated with chitin utilization. Because chitin is absent in mammalian hosts, its role during infection remains unclear. Here, we investigate the functional potential of the *S.* Typhimurium *chb* operon, with particular focus on ChbF, annotated as a 6-phospho-β-glucosidase. Together, our findings support a possible model in which the *S.* Typhimurium *chb* operon functions as a glycan-processing system that targets host-derived LacNAc-containing glycans and establishes a framework for future functional characterization.

**Figure 1. S. Typhimurium chb operon analysis and structural modeling of StChbF:** **A** ) Genetic organization of the *chb* operon in *S.* Typhimurium. **B** ) AlphaFold model for *S.* Typhimurium ChbF *(* StChbF *).* 6-Phospho-glucose from the 6-phospho-β-glucosidase from *Thermatoga maritima* (TmGH4: PDB ID 7CTM) is modeled into the active site and shown as a yellow stick. The loop spanning residues 231-240 are highlighted in red. **C** ) Sequence logo for residues found in the loop region. Comparison of the **D** ) NAD ^+^ binding site, **E** ) metal binding site, and **F** ) -1 subsite between StChbF (gray) and TmGH4 (green). Active site residues are labeled with StChbF first, followed by TmGH4. The manganese ion is shown as a purple sphere. 6-Phospho-glucose is shown as a yellow stick with the carbon 4 labeled as C4. **G** ) -1 subsite for StChbF with 6-phospho-galactose modeled as a cyan stick with the carbon 4 labeled as C4. **H** ) Surface representation of the -1 subsite for StChbF with 6-phospho-galactose modeled using Pymol as a cyan stick with the carbon 4 labeled as C4. Nitrogens are shown in blue, oxygens in red, sulfur is dark yellow, and phosphorus in orange. **I** ) Proposed LacNAc degradation pathway for *S.* Typhimurium *.* Galactose and glcNAc are represented as a yellow circle and blue rectangle, respectively.

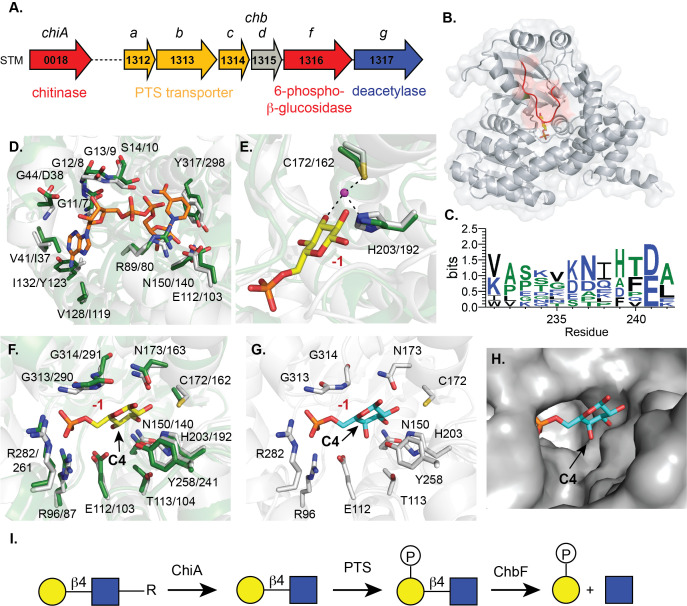



## Description

*Salmonella enterica* serovar Typhimurium ( *S.* Typhimurium) is an enteric pathogen with the capability of infecting both humans and animals. Infection begins with the ingestion of contaminated food or water where the bacteria reach the intestinal epithelium and cause gastrointestinal disease. A defining feature of *S.* Typhimurium pathogenesis is the ability to survive and replicate within host macrophages, by deploying specialized virulence factors and metabolic systems (Haraga et al., 2008).

Several studies have sought to identify *S.* Typhimurium virulence factors (Harvey et al., 2011; Hautefort et al., 2008; Lawley et al., 2006; Niemann et al., 2011). Although secretion systems are widely recognized as central contributors to *S.* Typhimurium pathogenesis (Ibarra & Steele-Mortimer, 2009), additional factors also play important roles. One such factor is ChiA, which has been shown to be upregulated during macrophage and epithelial infection (Eriksson et al., 2003; Hautefort et al., 2008), contribute to bacterial adhesion and invasion (Krone et al., 2023), and play a key role in dissemination from the small intestine (Devlin et al., 2022).

ChiA is a secreted chitinase belonging to glycoside hydrolase family 18 (GH18) (Larsen et al., 2011). Chitinases classically hydrolyze chitin and chitooligosaccharides, which are composed of repeating *N* -acetylglucoasmine (GlcNAc) units (Horn et al., 2006). Because mammals do not synthesize chitin, ChiA’s role as a virulence factor was not immediately apparent. However, recombinant ChiA from *S.* Typhimurium has also been shown to remove *N* -acetyllactosamine (LacNAc; Gal-GlcNAc) from glycoconjugates (Larsen et al., 2011). Since LacNAc motifs are commonly found in complex glycans on cell surfaces, this activity has been proposed to contribute to ChiA-mediated virulence by remodeling host surface glycans (Devlin et al., 2022).

Interestingly, ChiA functions as the first enzyme in a chitin degradation pathway in other organisms, such as *E. coli* (Walter et al., 2021). The remaining enzymes in this pathway are encoded within a *chb* operon. This operon typically includes a phosphoenolpyruvate-dependent phosphotransferase system (PTS) that phosphorylates and takes up ChiA-derived products. Until recently, it was thought that the intracellular 6-phospho-β-glucosidase, or glycoside hydrolase from family 4 (ChbF), would then depolymerize the internalized phosphorylated chitin disaccharides (GlcNAc6P-GlcNAc) into monosaccharides. However, the presence of a deacetylase ( *chbG)* in the *chb* operon warranted further investigation. Recently, it was found that ChbF is not active on GlcNAc6P-GlcNAc and that it requires ChbG to remove the *N* -acetyl group from the non-reducing end converting it to glucosamine 6-phosphate disaccharide (GlcN6P-GlcNAc), which can then be cleaved by ChbF (Walter et al., 2021).

The *S.* Typhimurium genome also contains an intact *chb* operon ( **Figure 1A** ). Notably, *chbF* and *chbG* is also upregulated during macrophage and epithelial infection, similar to *chiA* , suggesting a role in pathogenesis beyond remodeling of host surface glycans (Eriksson et al., 2003; Hautefort et al., 2008). Together, these observations suggest that *S.* Typhimurium may possess a coordinated ChiA–chb system that enables the processing and import of host LacNAc-containing glycans important for virulence. Although ChbF has not been shown to act on the phosphorylated LacNAc disaccharides that would be imported into the bacterium, we hypothesize that *S.* Typhimurium ChbF (StChbF) can utilize this substrate. In this study, we use bioinformatic approaches to assess possible alternative functions of StChbF and propose a potential LacNAc degradation pathway that may contribute to *S.* Typhimurium virulence.

*S.* Typhimurium ChbF (StChbF: STM1316; Uniprot ID Q8ZPU2) belongs to glycoside hydrolase family 4 (GH4), which have a unique NAD-dependent mechanism (Yip et al., 2007; Yip & Withers, 2006). Members of the GH4 family exhibit diverse activities, including α-glucosidase, α-galactosidase, α-glucuronidase, 6-phospho-α-glucosidase, and 6-phospho-β-glucosidase activity (Drula et al., 2022). Since StChbF is encoded within the *chb* operon ( **Figure 1A** ), which is associated with the degradation of β-linked chitin substrates, it likely functions as a 6-phospho-β-glucosidase. Sequence analysis further supports this assignment, as StChbF shares 90% amino acid identity with the characterized ChbF from *E. coli* (Thompson et al., 1999; Walter et al., 2021). Together, its strong sequence conservation and genomic context suggest that StChbF primarily participates in chitin-derived carbohydrate metabolism.

To further explore the potential of StChbF to act on other 6-phospho-β-linked disaccharides, such as LacNAc, we first examined its AlphaFold-predicted structure. The model adopts an NAD(H) binding Rossmann fold ( **Figure 1B** ) (Varrot et al., 2005). As expected, Foldseek analysis (Van Kempen et al., 2024) identified the closest experimentally validated structural homologs as 6-phospho-β-glucosidases from *Thermatoga maritima* (TmGH4: PDB ID 7CTM, RMSD 1.01 Å over 2030 atoms) (Mohapatra & Manoj, 2019, 2021; Varrot et al., 2005) and *Geobacillus stearothermophilus* (GsGH4: unpublished PDB ID 5C3M, RMSD 0.822 Å over 2170 atoms). A notable difference is a loop region spanning residues 231-240 that is present in the StChbF model and extends into the active site ( **Figure 1B** ). This region exhibits low model confidence and is absent in the available GH4 crystal structures. In addition, we generated a sequence logo (Crooks et al., 2004), and observed that this region does not appear to be conserved amongst characterized GH4 enzymes with 6-phospho-β-glucosidase activity ( **Figure 1C** ). Because these homologous structures are either holo or product-bound structures (with ligand occupying the -1 subsite), this loop may become ordered upon substrate binding and contribute to specificity at the +1 subsite. Further experiment evidence will be required to test this hypothesis.

We next compared the ligand-binding sites of StChbF and TmGH4. First, we examined the NAD ^+^ and metal-binding sites and, not surprisingly, found that they are almost completely conserved ( **Figures 1D and 1E** ). We also compared the -1 subsite, which binds 6-phosphoglucose, with the TmGH4 structure bound to the product 6-phosphoglucose at that site. We observed complete conservation of the -1 subsite architecture ( **Figure 1F** ). In StChbF, the -1 subsite is formed by the side chains of C172, N150, H203, Y258, T113, E112, R96, R282, and N173. This further suggests that StChbF has similar substrate specificity to TmGH4 and other ChbF enzymes, specifically on GlcN6P-GlcNAc (Mohapatra & Manoj, 2019; Walter et al., 2021).

To assess whether StChbF could accommodate a potential 6-phosphoLacNAc substrate (Gal6P-GlcNAc), we modeled 6-phosphogalactose in the -1 subsite. To accomplish this, we superimposed 6-phosphogalactose onto the 6-phosphoglucose bound in the -1 subsite of the TmGH4 structure ( **Figure 1G** ). The sole difference between 6-phosphogalactose and 6-phosphoglucose is the configuration of the C4 hydroxyl group. In the TmGH4 structure, the C4 hydroxyl of 6-phosphoglucose does not appear to form direct interactions with active site residues. When modeled as 6-phosphogalactose, the C4 hydroxyl does not appear to introduce steric clashes with surrounding residues, indicating that the sugar may be accommodated in the active site ( **Figure 1H** ).

Although GH4 enzymes with 6-phospho-β-glucosidase activity are generally considered glucose-specific, precedent exists for dual substrate specificity in related glycosidases. Notably, some members of family 1 glycoside hydrolases have 6-phospho-β-glucosidase and 6-phospho-β-galactosidase activity (Honda et al., 2012; Plaza-Vinuesa et al., 2023). However, direct experimental evidence for 6-phospho-β-galactosidase activity within the GH4 family would require biochemical validation.

Based on biochemical characterization of *S.* Typhimurium ChiA, transcriptomic analyses, and structural modeling of ChbF, we propose a potentially promiscuous ChiA–chb pathway that enables *S.* Typhimurium to degrade host-derived LacNAc-containing glycans ( **Figure 1I** ). In this proposed pathway, secreted ChiA cleaves a terminal LacNAc unit, releasing a LacNAc disaccharide (Larsen et al., 2011). The resulting disaccharide would then be imported via a PTS transporter and phosphorylated during uptake. This transporter could correspond to the Chb PTS system or an as-yet unidentified PTS system. Once internalized, ChbF would hydrolyze the phosphorylated LacNAc disaccharide to produce 6-phosphogalactose and GlcNAc. Notably, this proposed pathway would not require ChbG, as 6-phosphogalactose lacks an N-acetyl group.

Collectively, transcriptomic analyses indicate that *S.* Typhimurium ChiA and the *chb* operon are upregulated during infection (Eriksson et al., 2003; Hautefort et al., 2008), suggesting a potential role in virulence. Although this system is classically associated with chitin degradation, chitin is not present in animal cells. Notably, *S.* Typhimurium ChiA has been shown to act on the alternative substrate LacNAc (Larsen et al., 2011), which is abundant on the surface of human cells, including macrophages. Structural modeling further supports that *S.* Typhimurium ChbF may accommodate 6-phosphogalactose in the −1 subsite, raising the possibility that it can process imported phospho-LacNAc. Together, these observations support a revised model in which the *S. Typhimurium* ChiA–chb system degrades host-derived LacNAc, potentially contributing to virulence, and provide a framework for future biochemical validation.
